# *Brevibacillus borstelensis* and *Streptomyces albogriseolus* have roles to play in degradation of herbicide, sulfosulfuron

**DOI:** 10.1007/s13205-016-0562-z

**Published:** 2016-11-16

**Authors:** Ridhima Arya, Navnit Kumar Mishra, Anil K. Sharma

**Affiliations:** Department of Biotechnology, Maharishi Markandeshwar University, Mullana, (Ambala), Haryana 133207 India

**Keywords:** Sulfosulfuron, *Brevibacillus borstelensis*, *Streptomyces albogriseolus*, Degradation, Metabolites

## Abstract

Use of herbicides, in particular sulfosulfuron, at more than recommended doses has raised major concerns about the health hazards for animals and humans. In the present study, isolation of sulfosulfuron-degrading *Brevibacillus borstelensis* and *Streptomyces albogriseolus* from the field soils in the northwestern region of India was carried out where the use of sulfosulfuron is predominant, and further assessed for their potential to degrade sulfosulfuron individually and together in a consortium form under lab conditions. Concentration of sulfosulfuron was reduced from 10 to 7.72 µg/ml in 12 h to 5.13 µg/ml in 20 h by *B. borstelensis* and the metabolites detected by LCMS–MS were aminopyrimidine and a rearranged amine in 12 and 20 h of growth. Similarly, *S. albogriseolus* reduced the concentration of sulfosulfuron from 10 to 6.74 µg/ml in 12 h to 6.62 µg/ml in 20 h with aminopyrimidine and a rearranged amine as metabolites. *B. borstelensis* and *S. albogriseolus* together also reduced the concentration of sulfosulfuron from 10 µg/ml in initial hour to 8.34 µg/ml in 12 h to 6.66 µg/ml in 20 h. Hence, *B. borstelensis* and *S. albogriseolus* provide a safer, inexpensive and effective way to bio-remediate the harmful and toxic sulfosulfuron from the environment if further explored at a larger field scale in near future.

## Introduction

The use of pesticides, herbicides and insecticides in agriculture has been significantly amplified in previous years which led to air, water and soil pollution in different regions of the world (Malik and Singh 1993; Walia et al. 1997; Chhonkar and Malik 2002; Sondhia 2008).

Herbicide-like isoproturon was used initially for controlling weeds in the crop but its use has led to expansion of resistance strains of *Phalaris minor* (Walia et al. 1997; Malik and Singh 1993). Nowadays, herbicides like clodinafop, sulfosulfuron and fenoxaprop-p-ethyl are being preferred for proficient weed control in wheat crop (Chhonkar and Malik 2002). The use of these herbicides in particular sulfosulfuron at more than recommended doses has raised an alarm about the health hazards for animals and humans because of its residues left in soil and crops following its application. Higher levels of residues have been detected in the surface soil that occurs by irrigation, thus posing a threat to groundwater pollution (Sondhia 2008). Effect of sulfosulfuron on *Phalaris minor*, along with its carryover effects to rotational crops has been studied previously and sulfosulfuron was found to be effective in controlling the weed but its carryover effects have been observed on maize and sorghum crops with an inhibition of 65–73 % of the biomass (Chhonkar et al. 2006).

Many adverse health effects have been found to be associated with sulfosulfuron toxicity in animals (Arnold et al. 2001). Similarly, in wheat crop, there was significant reduction in yield and kernel number per year due to residual sulfonylurea herbicide (Bahrampor and Ziveh 2013). Sulfosulfuron phytotoxicity has been observed in cucurbits and other plants grown in field soil after the harvest of wheat crop (Walia et al. 2000; Kaur et al. 2010). A sensitive and very fast analytical method was developed for simultaneous detection of 16 sulfonylurea herbicides including sulfosulfuron in surface water (Yan et al. 2011). Residues of the sulfosulfuron and their harmful effects are detected in crops including sunflower, canola, bean, soybean, lens, sorghum, pea, sugar beet, corn, barley, and sorghum (Hadizadeh 2010). Leaching potential of sulfosulfuron and its metabolite aminopyrimidine in two different soils under lab conditions was studied and the leachates collected from the sandy clay soil and clay loam soil were shown to have high percentages of residues of sulfosulfuron as well as aminopyrimidine (Loganayagi and Ramesh 2013).

Sulfonylurea herbicides were reported to be very much persistent in the environment. These herbicides were used to control a wide range of annual and perennial narrow and broadleaf weeds. The main pathways for sulfonylurea metabolism in the soil have been reported to be chemical hydrolysis and microbial degradation (Brown 1990; Bossi et al. 1999). A number of cases have been reported for detection of sulfosulfuron in soil, fruits, cereals and vegetables and only a few studies are accessible about the microbes degrading them. Keeping the above facts in view, the present study was designed to isolate and characterize sulfosulfuron degrading microorganisms from the field soils where use of sulfosulfuron has been predominant and further screening of their potential to degrade sulfosulfuron individually and together in a consortium form under laboratory conditions.

## Materials and methods

For isolation, soil samples had been collected from wheat fields of northern regions of India. Upper layer of the leaves and debris were removed and 6 inches deep soil near to the root of wheat cereal plants (rhizosphere part) was collected. Serial dilution method was used for enumeration of total number of microbes and Nutrient Agar was used for the growth of microbes. Microorganisms were selected by enrichment culture technique with sulfosulfuron (25 µg/ml) addition to Jensen’s medium. The number of microbes was enumerated as colony-forming units/ml (CFU/ml). Specific colony characteristics like colour, shape, size, hardness, appearance, texture, pigmentation and diameter were observed. Optimization of strain was done using standard procedures. For selection, the selected colonies were re-plated on agar medium containing sulfosulfuron (25 µg/ml) and further checked for growth. Serial dilutions of the two isolates (10^−1^, 10^−2^, 10^−3^, 10^−4^) were spotted onto the media plates containing different concentrations of sulfosulfuron (25, 50 and 500 µg/ml, respectively). Cells were stained according to classical Gram’s staining and endospore staining as well.

Further morphological, biochemical and physiological analysis of the two isolates was carried out at Microbial Type Culture Collection, IMTECH, Chandigarh, India. Culture samples were collected at initial time point (0 h), 12 and 20 h growth of *Brevibacillus borstelensis* and *Streptomyces albogriseolus* grown in Jensen’s medium containing sulfosulfuron (10 µg/ml) at 37 °C under shaking conditions. Assessment of the metabolites/degradation products of sulfosulfuron from the above culture samples was studied by liquid chromatography mass spectrometry (LCMS–MS) and HPLC/MS analysis carried at Punjab Biotechnology Incubator, an NABL-accredited Agri and Food testing lab, SAS Nagar (Mohali), Punjab, India.

Using both the microorganisms together, a microbial consortium was developed. Culture samples were collected at initial 0, 12 and 20 h growth of *B. borstelensis* and *S. albogriseolus* grown together in Jensen’s medium containing sulfosulfuron (10 µg/ml) at 37 °C under shaking conditions. The culture samples were subjected to analysis at Punjab Biotechnology Incubator, Punjab, India, for LC–MS/MS analysis.

## Results

As we know, the remediation of the environment and soil is the need of the hour for removal of unwanted chemical pesticides to protect the environment from their ill effects. The present study was done to investigate some novel microbial species for restoring the environment from the ill effects of sulfosulfuron by degradation or removal of this weedicide. The isolated bacterium *B. borstelensis* was found to grow well in the pH range of 6–9 and the maximum growth was obtained at pH 7. *B. borstelensis* displayed growth in the temperature range of 30–40 °C and growth optima was observed at 37 °C. Another bacterium *S. albogriseolus* was observed to grow well in the pH range 7–9 and maximum growth was obtained at pH 8. *S. albogriseolus* displayed growth at temperature conditions ranging from 30 to 40 °C and maximal growth was observed at 37 °C. Both strains were found to grow well at higher concentration of the pesticide sulfosulfuron, and isolated colonies were obtained at higher dilutions of the strains (Table 1).

**Table 1:** Effect of different concentrations of sulfosulfuron on the growth of *Brevibacillus borstelensis* and *Streptomyces albogriseolus*

Isolate	Conc. of pesticide-Sulfosulfuron (µg/ml)	Neat	10^−1^	10^−2^	10^−3^	10^−4^
		Growth of *Brevibacillus borstelensis* (no. of colonies)				
*Brevibacillus borstelensis*	25	Confluent	Confluent	Confluent	53	29
	50	Confluent	Confluent	55	42	26
	500	Confluent	Confluent	46	28	20

	Sulfosulfuron (µg/ml)	Growth of *Streptomyces albogriseolus* (no. of colonies)				
*Streptomyces albogriseolus*	25	Confluent	Confluent	Confluent	19	26
	50	Confluent	Confluent	Confluent	23	19
	500	Confluent	Confluent	Confluent	20	15

**Fig. 1:**
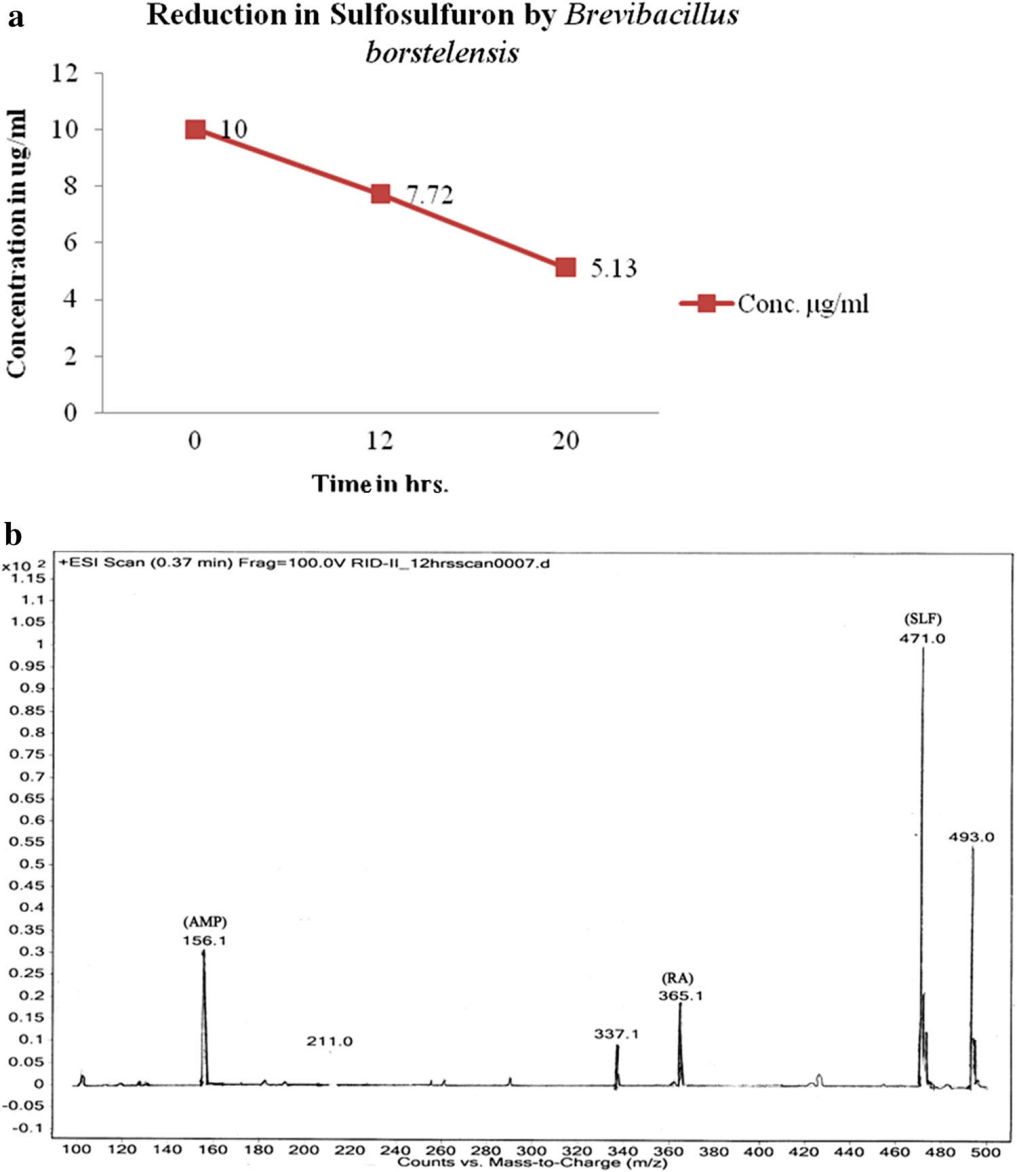
**a** Reduction in sulfosulfuron concentration by *Brevibacillus borstelensis.* **b** Sulfosulfuron metabolites detection in 12-h growth sample of *Brevibacillus borstelensis*

**Fig. 2:**
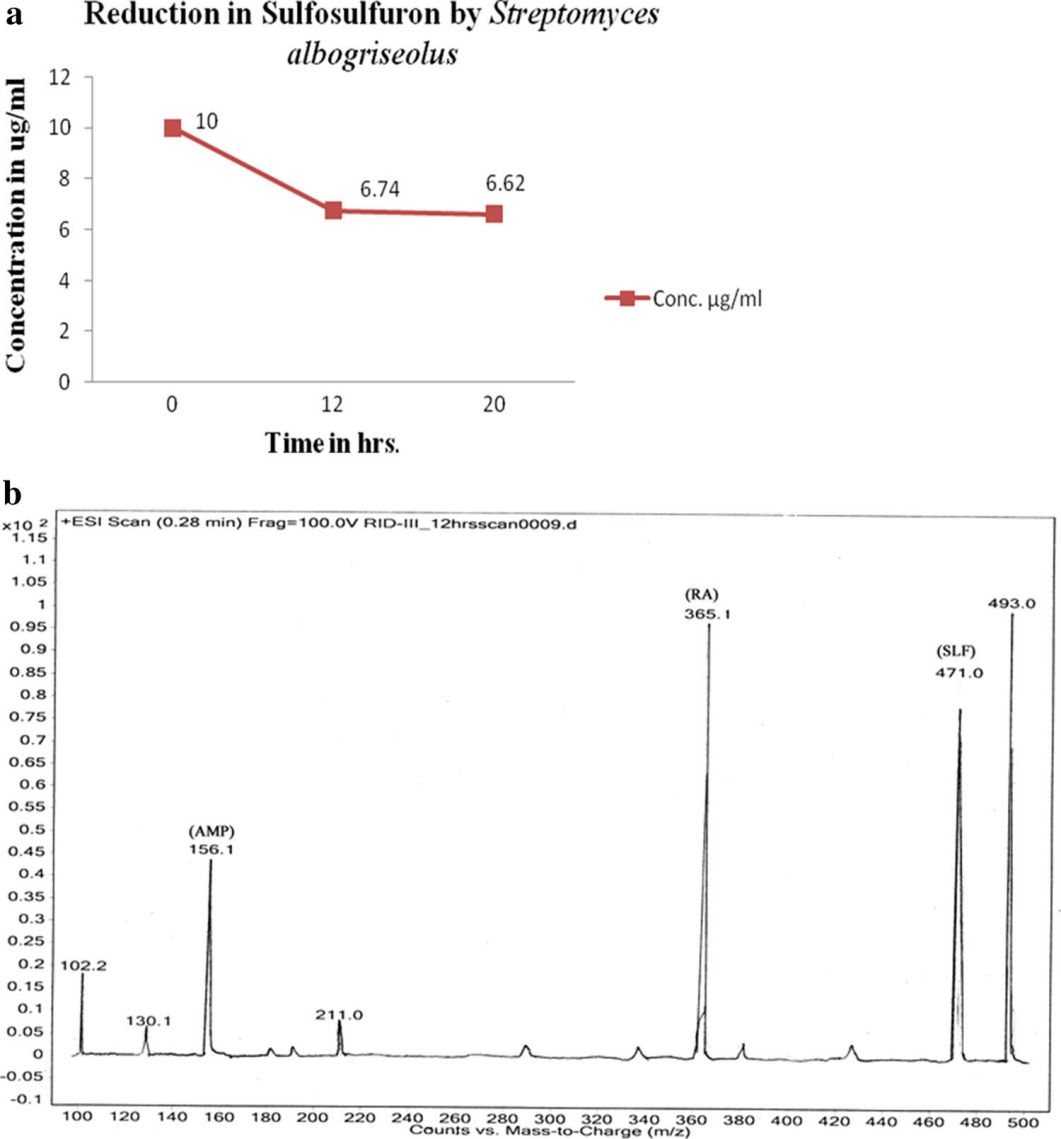
**a** Reduction in sulfosulfuron concentration by *Streptomyces albogriseolus.* **b** Sulfosulfuron metabolites detection in 12-h growth sample of *Streptomyces albogriseolus*

**Fig. 3:**
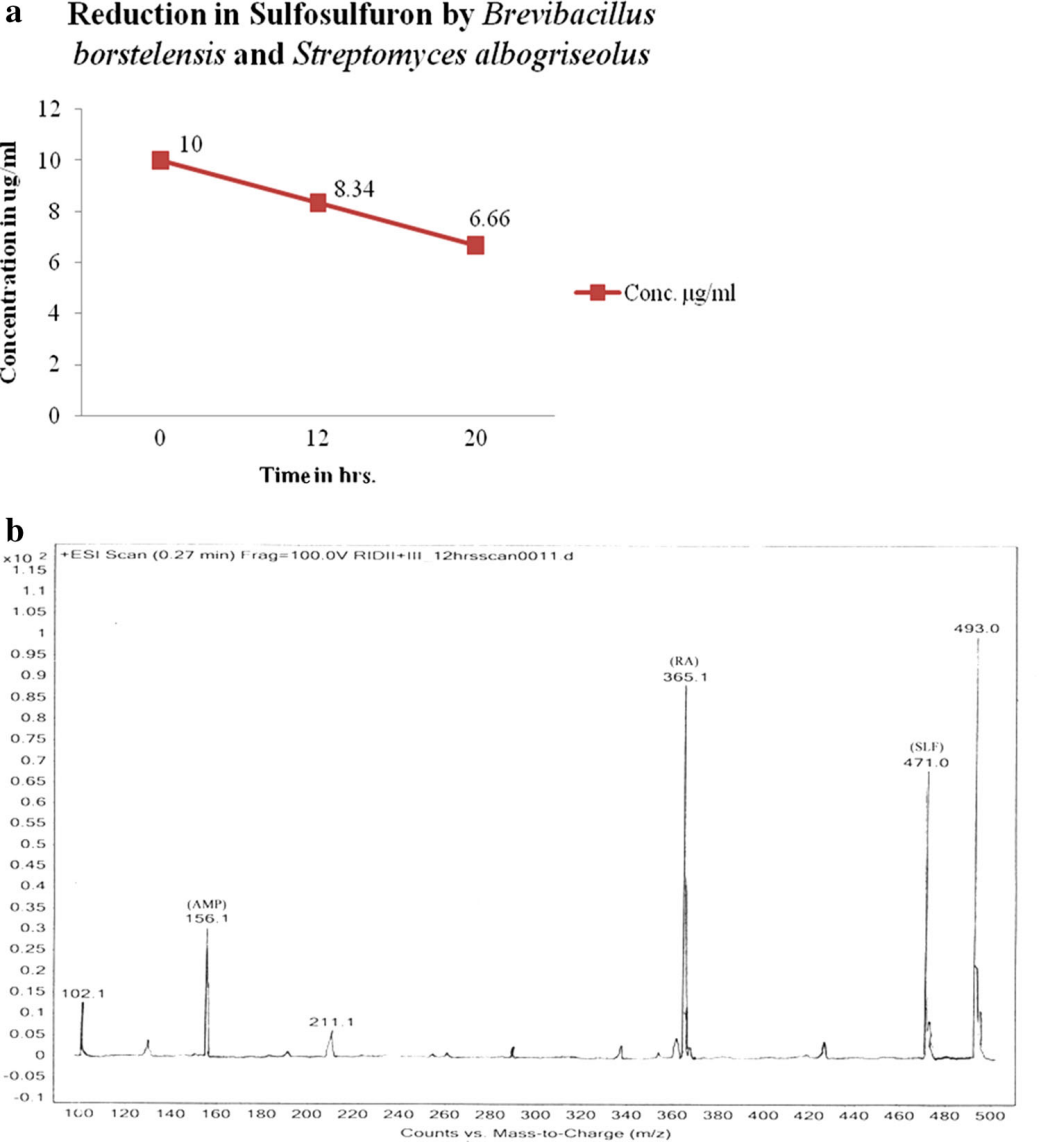
**a** Reduction in sulfosulfuron concentration by *Brevibacillus borstelensis* and *Streptomyces albogriseolus* grown together as consortia. **b** Sulfosulfuron metabolites detection in 12-h growth sample of consortium containing both isolates *Brevibacillus borstelensis* and *Streptomyces albogriseolus*

Concentration of sulfosulfuron was reduced from 10 to 7.72 µg/ml in 12 h to 5.13 µg/ml in 20 h by *B. borstelensis* (Fig. 1a). Metabolites detected by LCMS–MS studies include aminopyrimidine and a rearranged amine in 12 and 20 h of the growth (Fig. 1b). Similarly, *S. albogriseolus* reduced the concentration of sulfosulfuron from 10 to 6.74 µg/ml in 12 h to 6.62 µg/ml in 20 h (Fig. 2a). Metabolites aminopyrimidine and a rearranged amine were detected by LCMS–MS in 12 and 20 h growth (Fig. 2b). The microbial consortia containing *B. borstelensis* and *S. albogriseolus* reduced the concentration of sulfosulfuron from 10 µg/ml in initial hour to 8.34 µg/ml in 12 h to 6.66 µg/ml in 20 h (Fig. 3a). Metabolites detected by LCMS–MS were aminopyrimidine and a rearranged amine in 12 h and 20 h growth of the consortia (Fig. 3b).

Both *B. borstelensis* and *S. albogriseolus* have the capability of successfully degrading sulfosulfuron within 10–20 h of growth. However, degradation was nearly the same in the microbial consortia form. These microbial strains have the ability of degrading sulfosulfuron residues in the soil environment which could be further explored by understanding degradation mechanisms at the genetic level. Efforts may further lead to enhancement of the potential of microorganisms to effectively degrade the pesticides, hence acting as valuable bioremediation agents in near future.

## Discussion

**Fig. 4:**
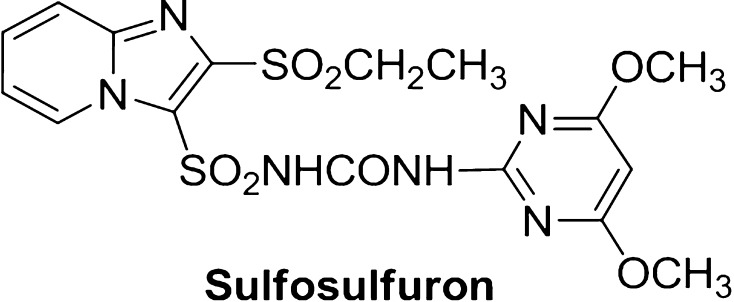
The IUPAC name of sulfosulfuron is 1-(4, 6-dimethoxypyrimidin-2-yl)-3-(2-ethylsulfonylimidazo [1, 2-a] pyridine-3-ylsulfonyl) urea. The molecular formula of sulfosulfuron is C_16_H_18_N_6_O_7_S_2_

A variety of microorganisms in the soil act as the scavengers of sulfosulfuron (Fig. 4) and is degradation products have been identified as 1-(2-ethylsulfonylimidazo[1,2-a]pyridine)-3-(4,6-dimethoxypyramidin-2-yl), 1-(2-ethylsulfonylimidazo [1,2-a]pyridine)-3-sulfonamide and 4,6-dimethoxy-2-aminopyramidine (Sondhia and Singhai 2008). Breakdown of pesticides could occur by plants, animals, soil, water and UV radiations, but degradation by microbes, especially fungi and bacteria, is foremostly important (Arya and Sharma 2014a, b, 2015, 2016).

Microbial degradation prevails in neutral to basic soils while in soils with lower pH, the hydrolytic degradation of the compounds is dominant (Menne and Berger 2001; Brar et al. 2006a, b; Sarmah and Sabadie 2002). The photocatalytic degradation of five sulfonylurea herbicides viz. chlorosulfuron, nicosulfuron, flazosulfuron, triasulfuron and sulfosulfuron was studied and their degradation followed first order kinetics and none of the pesticides were detected after 120 min of illumination except chlorosulfuron (Fenoll et al. 2012). Sondhia and Singhai (2008) have shown in their study, the cleavage of the sulfonylurea bridge to form 1-(2-ethylsulfonylimidazo [1, 2-a] pyridine)-3-sulfonamide and 4, 6-dimethoxy-2-aminopyramidine. Ramesh et al. (2007) through LCMS/MS analysis on water and fish samples, explored the presence of metabolites, ethyl sulfone, aminopyrimidine, desmethyl sulfosulfuron, sulphonamide, guanidine and a rearranged amine.

The stability of sulfosulfuron has been analysed by (Saha and Kulshrestha 2002) in a controlled environment of temperature, pH and solvent as well its photo-stability after irradiation under sunlight. 1-(2-ethylsulfonylimidazo [1,2-a]- pyridin-3-yl-3-(4,6-dimethoxypyrimidin-2-yl) amine as degradation product was observed under alkaline conditions by Saha et al. (2003), and under acidic condition, it degraded to two metabolites, 1-(2-ethylsulfonylimidazo [1,2-a] pyridin)-3-sulfonamide and 4,6-dimethoxy-2-aminopyrimidine by cleavage of sulfonylurea bridge.

*Brevibacillus borstelensis* as well as *S. albogriseolus* in our study were found to reduce sulfosulfuron into aminopyrimidine and a rearranged amine due to the cleavage or opening of the sulfonylurea bridge of the sulfosulfuron as also discussed elsewhere (Sondhia and Singhai 2008; Saha et al. 2003). Similarly, consortium containing *B. borstelensis* and *S. albogriseolus* grown together reduced sulfosulfuron into aminopyrimidine and a rearranged amine which may have resulted because of the cleavage or opening of the sulfonylurea bridge of the sulfosulfuron (Saha et al. 2003; Saha and Kulshrestha 2002). Both *B. borstelensis* (Arya and Sharma 2014a, b) and *S. albogriseolus* (Arya et al. 2015) alone and together in consortia form (Arya and Sharma 2016) were also reported previously to degrade a fungicide, carbendazim to 2-aminobenzimidazole, 2-hydroxy benzimidazole and benzimidazole as well.

## Conclusions

Since bioremediation of harmful substances such as sulfosulfuron from the environment is a prerequisite to maintain the environmental inertia, we identified and characterized two microbial strains, i.e., *B. borstelensis* and *S. albogriseolus* which have the capability of effectively degrading sulfosulfuron within 10–20 h of growth although no synergistic effect was observed in the microbial consortia form. LCMS–MS analysis further detected aminopyrimidine and a rearranged amine as metabolites in 12 and 20 h of the microbial growth. However, the potential of these microbial strains to degrade sulfosulfuron needs to be further investigated at the genomic level to understand the effectivity of these strains as bioremediation agents.
